# Topological insights into breast cancer drugs: a QSPR approach using resolving topological indices

**DOI:** 10.3389/fchem.2025.1710442

**Published:** 2025-10-29

**Authors:** E. Pandeeswari, J. Ravi Sankar

**Affiliations:** Department of Mathematics, School of Advanced Sciences, Vellore Institute of Technology, Vellore, Tamil Nadu, India

**Keywords:** resolving set, metric dimension, resolving degree, resolving topological indices, regression models, QSPR study

## Abstract

**Introduction:**

Breast cancer, one of the most prevalent malignancies in women begins in the milk ducts or lobules and is divided into invasive and non-invasive variants. The kind stage and molecular features of the cancer determine the treatment strategy which may include surgery, chemotherapy, and targeted drugs. Early identification through screening is critical to increasing patient survival rates.

**Methods:**

In this study, we look at the efficacy of numerous breast cancer drugs, including Toremifene, Tucatinib, Ribociclib, Olaparib, Abemaciclib, Anastrozole, Letrozole, Thiotepa, Tamoxifen, and Megestrol Acetate. We investigate their chemical and physical properties, including molar volume (MV), polarizability (P), molar refractivity (MR), polar surface area (PSA), and surface tension (ST). We employ Quantitative Structure Property Relationship (QSPR) analytical approaches, including curvilinear regression and multiple linear regression (MLR), to model and predict the physicochemical properties of these medications by analyzing the impact of molecular descriptors on these properties.

**Results:**

A comparison of the two regression techniques is done to see how accurate their predictions are and to find the best way to model the data. Furthermore, resolving topological indices examines the relationship between molecular structure and therapeutic effectiveness.

**Discussion:**

The outcomes of these studies help to further our understanding of breast cancer treatments and the development of more focused and customized therapeutics.

## 1 Introduction

Chemical graph theory is an interdisciplinary study that uses principles from chemistry and graph theory to investigate the structural features of chemical molecules. Researchers can investigate molecular stability, reactivity, and spectrum features by portraying molecules as graphs with atoms as vertices and bonds as edges. This technique not only helps to comprehend complicated chemical processes, but it also makes it easier to develop novel materials and medications by shedding light on the links between molecular structure and function (Liu, 2022).

The investigation of resolving sets and metric dimensions in chemical graph theory provides important insights into the discovery and characterisation of molecular structures. A resolving set is a subset of vertices in a graph that can be uniquely recognized by its distances to the other vertices in the set. This idea is critical in understanding how distinct atoms within a molecule may be identified based on connectedness, which is required for predicting chemical behavior and reactivity. The metric dimension, which is defined as the smallest size of a resolving set for a given graph, measures the effectiveness of this identification method.

The concept of metric dimension in graph theory, proposed by (Slater, 1975), is closely related to the concept of a resolving set, which is a collection of vertices that uniquely identify all other vertices based on their distance (Harary and Melter, 1976). defined the metric dimension as the size of the smallest resolving set in a graph. This notion has implications in network theory and molecular graph analysis, where resolving sets aid in identifying chemical structures. Metric dimension and resolving sets remain significant techniques in graph theory, with applications in cheminformatics and structural biology. These significant materials continue to be important in the study of graph based models across a variety of scientific disciplines.

Degree based topological indices, which depend on vertex degrees in molecular networks, are widely used to predict chemical characteristics and biological activities (Randic, 1975). proposed the Randić index, which is defined as $R=\sum_{uv\in E}{\left(d_{u}d_{v}\right)}^{-1/2}$ (Gutman and Trinajstić, 1972). Zagreb indices are the basis for QSPR and QSAR research. The first Zagreb index is $M_{1}=\sum_{u\in V}d_{u}^{2}$ , and the second is $M_{2}=\sum_{uv\in E}d_{u}d_{v}$ . The hyper-Zagreb and modified Zagreb indices have been extended to increase their forecast accuracy in complex systems. The study utilizes multigraphs and topological indices (TIs) in QSPR/QSAR analysis of antiviral medications such as Lopinavir and Remdesivir, as well as multiple linear regression (MLR), to connect physicochemical qualities with biological activity, therefore improving knowledge of treatment efficacy against COVID-19 (P et al., 2024). In 2021, developed a new vertex degree known as the domination degree of v , which is based on dominance sets with certain properties. In Bommahalli Jayaraman and Siddiqui (2024), the authors explored the basic features of the dominance degree function and got accurate values. Zagreb dominance indices for several graph families. This study utilized QSPR models with topological indices to predict the physicochemical characteristics of AD medicines, resulting in more efficient drug design Sardar and Hakami (2024). This study employed degree-based topological indices and two novel Zagreb-type descriptors to assess the physicochemical parameters of kidney cancer medicines Mahboob et al. (2024). Regression analysis revealed excellent correlations with experimental data, indicating their predictive reliability.

Predicting the physicochemical characteristics of medicines using different regression models has been the subject of several papers. Linear regression models and degree-related topological indices are used to evaluate kidney cancer drugs. Havare recently used three regression models and degree-related metrics to assess cancer drugs (Havare, 2021). The characteristics of cancer drugs are closely connected, according to quadratic regression. Cancer characteristics, like molar volume, polarizability, and molar refractivity, are more strongly correlated than previously thought. Kirmani et al. identified 10 features of antiviral drugs by using 11 degree-related TIs (Kirmani et al., 2021). In 2021, Liu et al. studied the chemical structures of coronavirus treatments using 15 distinct indices (Liu and Singaraj, 2021). Rauf et al. examined the COVID-19 drug structure’s molar refractivity, polar surface area, and molar volume with basic and multiple linear regression (Rauf et al., 2023a; Rao et al., 2024). Similarly, regression models and topological indices are used to study the structures of many drugs (Kumar and Das, 2024). This work investigates the use of topological indices, namely, hydrogen representation, to predict the physicochemical features of TCA medications (Kour and Ravi Sankar, 2025). This study uses distance-based topological indices and QSPR analysis to analyze the physicochemical characteristics of tricyclic antidepressant medications, stressing their importance in structure-property prediction (Kour and Sankar, 2025). Machine learning with distance-based topological indices from hydrogen-depleted networks allows for reliable QSPR prediction of anticancer drug characteristics, which aids efficient drug development (Kour et al., 2024). In QSPR modeling, graph-theoretical descriptors have proven useful, as demonstrated by earlier research, such as our work on NSAIDs employing Degcity indices (Pandeeswari et al., 2025; Kara et al., 2025a) used topological polynomials and indices to analyze lung cancer medicines and found high connections with physicochemical attributes and prediction accuracy. These findings support the use of topological indices as credible descriptors in chemical graph theory. Similarly, Arockiaraj et al. (2025) used degree and neighborhood degree sum topological indices to analyze cancer drug structures, proving their great prediction abilities using QSPR models. These findings further support the use of topological indices as trustworthy descriptors in molecular property estimation (Hakeem et al., 2025). Topological modeling and QSPR analysis were utilized to forecast the physicochemical features of bioactive polyphenols. These results show that degree-based indices may successfully link molecular structure to physical characteristics, which aids medication design. Furthermore, Kara et al. (2025b) neighborhood eccentricity-based indices have been used to COVID-19 drugs, yielding good correlations with physicochemical parameters and confirming the use of topological descriptors in drug design. In our earlier study (Pandeeswari and Ravi Sankar, 2025), we used chemical graph theory to investigate the vertex and edge metric dimensions of several breast cancer drug structures in detail. This fundamental study establishes a formal framework for using metric dimension notions to define molecular structures and improve predictive modeling. This study also expands on previous research Sooryanarayana et al. (2022), such as the resolving topological indices created for standard networks and their use in silicate structures, by applying the approach to breast cancer drugs. Existing indices, such as the Zagreb indices and metric dimension ideas, serve as standards. The novelty is in using these indices for medicinal compounds and combining them with modern computational tools such as LR and MLR to improve predictive modeling. Collectively, these investigations demonstrate topological indices efficacy as trustworthy and cost-effective descriptors in chemical graph theory. In this work, we explore the use of resolving topological indices to examine the physicochemical properties of drugs used to treat breast cancer. These articles represent a link between mathematics and pharmaceuticals.

The purpose of this study is to investigate the possibility for resolving topological indices in the computational analysis of breast cancer medications. Resolving indices, developed from graph theory, offer new insights into molecule structures by capturing their topological characteristics. This study employs these indices along with QSAR/QSPR approaches to simulate important physicochemical properties of breast cancer drugs, which can serve as a basis for future studies aimed at predicting pharmacological efficacy. This work stresses the importance of mathematical modeling and computational approaches in aiding drug development and generating insights that may eventually lead to specific cancer treatment options.

To the best of our knowledge, this is the first systematic research that uses resolving topological indices in QSPR modeling of breast cancer drugs. By including these indicators into regression models, the current study not only demonstrates their predictive power, but also gives new perspectives on the structural determinants of breast cancer drug efficacy.

## 2 Preliminaries

This section introduces the fundamental ideas and terminologies used in the study of chemical graphs. This covers definitions for resolving sets, metric dimensions, and resolving degree-based topological indices, all of which are required to comprehend molecular graph structure analysis. Lemma 1 offers a theoretical basis for computing resolving degree-based topological indices. These indices are useful tools in molecular characterization, since they assist in predicting molecular behavior and bio activity.

### 2.1 Resolving set and metric dimensions in chemical graph

Let G represent a molecular graph, which is a simple, connected, and undirected graph where the vertex set V(G) corresponds to atoms and the edge set E(G) corresponds to chemical bonds. A resolving set $S=\left\{v_{1},v_{2},v_{3},\ldots ,v_{k}\right\}\subseteq V\left(G\right)$ satisfies the following:

1. S is an ordered subset of the atoms (vertices) in V(G) .
2. For each atom x∈V(G) , its representation vector with respect to S , defined as:

$$r\left(x|S\right)=\left(d\left(x,v_{1}\right),d\left(x,v_{2}\right),\ldots ,d\left(x,v_{k}\right)\right),$$

is unique. Here, $d\left(x,v_{k}\right)$ denotes the shortest path distance between x and $v_{k}$ , which corresponds to the minimum number of bonds traversed between the two atoms in the molecular graph G .

A resolving set with the minimum cardinality is called a *metric basis*, and the size of this metric basis is referred to as the *metric dimension* of the molecular graph G , denoted as dim(G) .

### 2.2 Degree related resolving topological indices of molecular graphs

• Sooryanarayana et al. (2022) The first resolving Zagreb indices of (G) represented by FRZI1(G) is defined as,

$$FRZI1\left(G\right)=\underset{a\in V}{\sum }d_{\beta }{\left(a\right)}^{2}$$ (1)

$$FRZI2\left(G\right)=\underset{ab\in E}{\sum }\left[d_{\beta }\left(a\right)+d_{\beta }\left(b\right)\right]$$ (2)

• Sooryanarayana et al. (2022) The second resolving Zagreb index of (G) represented by SRZI(G) is defined as,

$$SRZI\left(G\right)=\underset{ab\in E}{\sum }\left[d_{\beta }\left(a\right)\cdot d_{\beta }\left(b\right)\right]$$ (3)

• Sooryanarayana et al. (2022) Resolving hyper Zagreb index of (G) represented by RHM(G) is defined as,

$$RHM\left(G\right)=\underset{ab\in E}{\sum }{\left[d_{\beta }\left(a\right)+d_{\beta }\left(b\right)\right]}^{2}$$ (4)

• Sooryanarayana et al. (2022) Resolving forgotten index of (G) represented by RF(G) is defined as,

$$RF\left(G\right)=\underset{ab\in E}{\sum }\left[d_{\beta }{\left(a\right)}^{2}+d_{\beta }{\left(b\right)}^{2}\right]$$ (5)

Lemma 1 Sooryanarayana et al. (2022) For every vertex v of a connected graph G, $\beta \left(G\right)\leq d_{\beta }\left(v\right)\leq \beta \left(G\right)+1$ , and $d_{\beta }\left(v\right)=\beta \left(G\right)$ iff there is a metric basis containing v.

### 2.3 Remark

In view of Lemma 1, the above Equations 1–5 can be written as.

$$FRZI_{1}\left(G\right)=\eta {\left(\beta \left(G\right)\right)}^{2}+\left(|V\left(G\right)|-\eta \right){\left(\beta \left(G\right)+1\right)}^{2}$$ (6)

$$FRZI_{2}\left(G\right)=2|E\left(G\right)|\beta \left(G\right)+\left(\xi_{1}+2\xi_{2}\right)$$ (7)

$$SRZI\left(G\right)=|E\left(G\right)|{\left(\beta \left(G\right)\right)}^{2}+\left(\xi_{1}+2\xi_{2}\right)\beta \left(G\right)+\xi_{2}$$ (8)

$$RHM\left(G\right)=4\beta {\left(G\right)}^{2}|E\left(G\right)|+4\beta \left(G\right)\left(\xi_{1}+2\xi_{2}\right)+\left(\xi_{1}+4\xi_{2}\right)$$ (9)

$$RF\left(G\right)=2\beta {\left(G\right)}^{2}|E\left(G\right)|+2\beta \left(G\right)\left(\xi_{1}+2\xi_{2}\right)+\left(\xi_{1}+2\xi_{2}\right)$$ (10)

Where

$$\begin{array}{ll} \eta & =\left|\left\{u:d_{\beta }\left(u\right)=\beta \left(G\right)\right\}\right|\\ \xi_{1} & =\left|\left\{e=uv\in E\left(G\right):d_{\beta }\left(u\right)=\beta \left(G\right),d_{\beta }\left(v\right)=\beta \left(G\right)+1\right\}\right|\\ \xi_{2} & =\left|\left\{e=uv\in E\left(G\right):d_{\beta }\left(u\right)=d_{\beta }\left(v\right)=\beta \left(G\right)+1\right\}\right| \end{array}$$

Theorem 1 Let G be the non-trivial connected molecular graph of the drug Toremifene. The resolving degree-based topological indices of G are:

$$FRZI_{1}\left(G\right)=599,\;FRZI_{2}\left(G\right)=284,\;SRZI\left(G\right)=651,\;RHM\left(G\right)=2618,\;RF\left(G\right)=1316.$$

Proof. Let G(V,E) be the molecular graph of Toremifene, where G contains 29 vertices (atoms) and 31 edges (bonds).

Now we define, The *resolving degree* of a vertex u , denoted by $d_{\beta }\left(u\right)$ , is defined as the minimum cardinality of a resolving set of G that contains the vertex u .

Let S be the metric basis of G . By Lemma 1, the following hold: $d_{\beta }\left(u\right)=\beta \left(G\right)$ for all u∈S and $d_{\beta }\left(u\right)\leq \beta \left(G\right)+1$ for all $u\in S^{c}$ , where $S^{c}=V\left(G\right)\backslash S$ .

For the graph G , we have:

$$\beta \left(G\right)=|S|=4,\;d_{\beta }\left(u\right)=\beta \left(G\right)=4\;\text{for all vertices }u\in S.$$

We calculate the following quantities:

$$\begin{array}{ll} \eta & =\left|\left\{u:d_{\beta }\left(u\right)=4\right\}\right|=14\\ \xi_{1} & =\left|\left\{e=uv\in E\left(G\right):d_{\beta }\left(u\right)=\beta \left(G\right),d_{\beta }\left(v\right)=\beta \left(G\right)+1\right\}\right|=14\\ \xi_{2} & =\left|\left\{e=uv\in E\left(G\right):d_{\beta }\left(u\right)=d_{\beta }\left(v\right)=\beta \left(G\right)+1\right\}\right|=11 \end{array}$$

Substituting the above values into the Equations 6‐10 for resolving degree based topological indices, we get:

$$\begin{array}{ll} FRZI_{1}\left(G\right) & =4{\left(4\right)}^{2}+\left(29-14\right){\left(5\right)}^{2}=599\\ FRZI_{2}\left(G\right) & =\left(2\right)\left(31\right)\left(4\right)+\left(14+22\right)=284\\ SRZI\left(G\right) & =\left(31\right){\left(4\right)}^{2}+\left(14+22\right)\left(4\right)+11=651\\ RHM\left(G\right) & =\left(4\right){\left(4\right)}^{2}\left(31\right)+\left(4\right)\left(4\right)\left(14+22\right)+\left(14+44\right)=2618\\ RF\left(G\right) & =2{\left(4\right)}^{2}\left(31\right)+\left(2\right)\left(4\right)\left(14+22\right)+\left(14+22\right)=1316. \end{array}$$

Thus, the resolving degree-based topological indices of G are as stated in the theorem.

Similarly, for other well-known breast cancer drugs, the corresponding graph invariants are calculated and presented in Tables 1, 2.

**TABLE 1:** Graph invariants for different breast cancer drugs.

Drugs	|V(G)|	|E(G)|	β(G)	η	$\xi_{1}$	$\xi_{2}$
Tucatinib	36	41	3	11	10	26
Ribociclib	32	36	4	14	12	17
Olaparib	32	36	3	8	10	23
Abemaciclib	37	41	3	10	10	27
Anastrozole	22	23	5	12	8	10
Letrozole	22	24	3	12	10	7
Thiotepa	11	13	3	6	6	4
Tamoxifen	28	30	4	14	14	10
Megestrol Acetate	28	31	4	12	9	17

**TABLE 2:** Obtained values of the resolving degree-based topological indices of breast cancer drugs.

Drugs	$FRZI_{1}\left(G\right)$	$FRZI_{2}\left(G\right)$	SRZI(G)	RHM(G)	RF(G)
Toremifene	599	284	651	2,618	1,316
Tucatinib	499	308	581	2,334	1,172
Ribociclib	674	334	777	3,120	1,566
Olaparib	456	272	515	2070	1,040
Abemaciclib	522	310	588	2,362	1,186
Anastrozole	660	258	725	2,908	1,458
Letrozole	268	168	295	1,190	600
Thiotepa	134	92	163	658	332
Tamoxifen	574	274	626	2,518	1,266
Megestrol Acetate	592	291	685	2,749	1,379

## 3 Materials and methods

**FIGURE 1:**
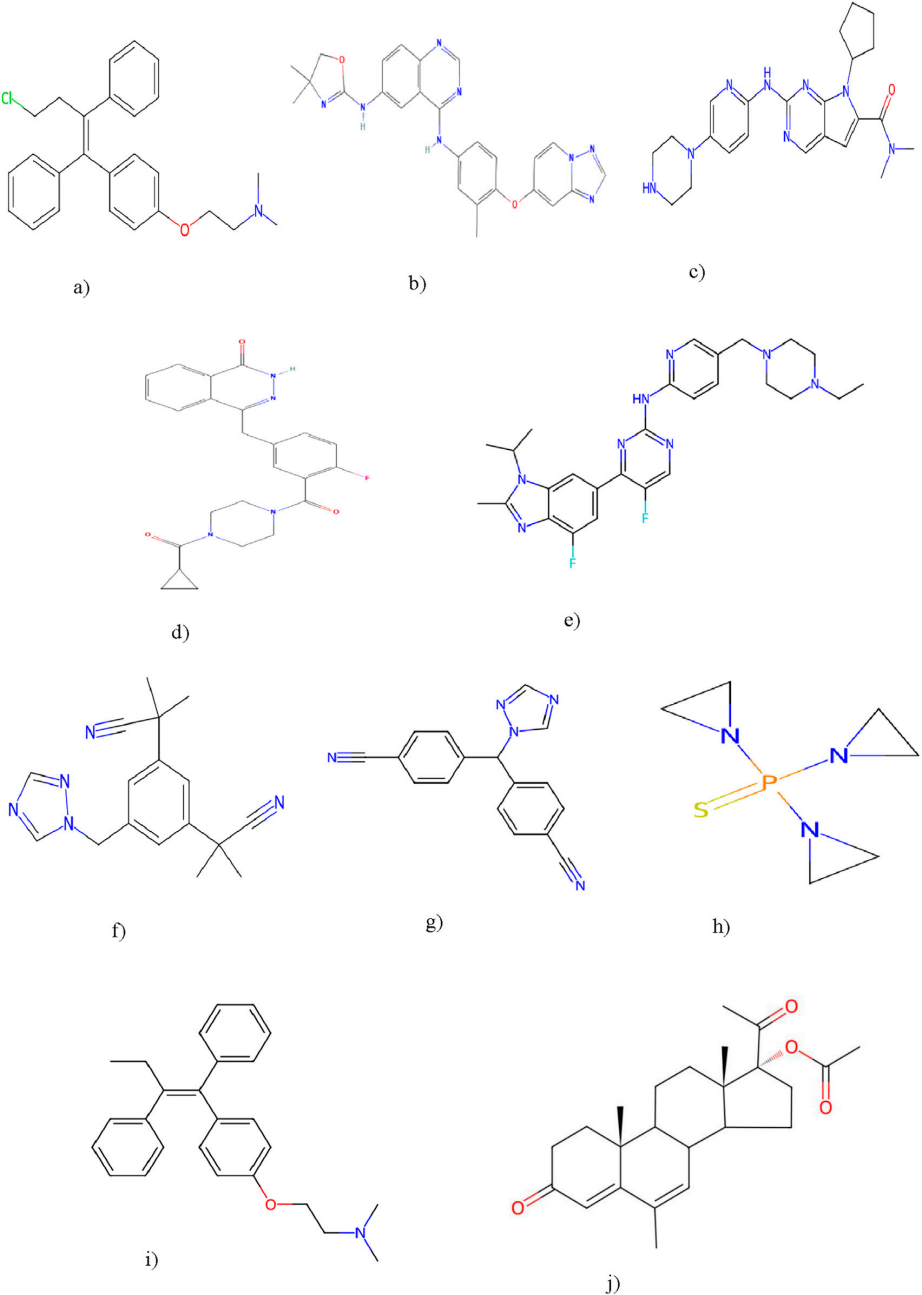
Breast cancer drugs: Toremifene, Tucatinib, Ribociclib, Olaparib, Abemaciclib, Anastrozole, Letrozole, Thiotepa, Tamoxifen, Megestrol Acetate. **(a)** Toremefine. **(b)** Tucatinib. **(c)** Ribociclib. **(d)** Olaparib. **(e)** Abemaciclib. **(f)** Anastrozole. **(g)** Letrozole. **(h)** Thiotepa. **(i) **Tamoxifen. **(j) **Megestrol acetate.

Resolving degree-based topological indices (RTIs) and statistical analysis are the two types of computations used in this study. ChemSpider provides the experimental findings, while JMP software and Excel are used for the statistical analysis. We can gain a deeper comprehension of chemical structures and behavior by employing these techniques and tools. This work uses resolving degree-based topological indices to analyze the chemical structures of drugs used to treat breast cancer. These indices of QSPR analysis are discussed and the results show a striking relationship with the physical characteristics of the chemical compounds used to treat breast cancer. This study focuses on ten drugs: Toremifene, Tucatinib, Ribociclib, Olaparib, Abemaciclib, Anastrozole, Letrozole, Thiotepa, Tamoxifen, and Megestrol Acetate. Figure 1 illustrates the chemical structures of these compounds. The particular physicochemical properties of breast cancer drugs are included in Table 3, which also provides helpful details regarding the molecular structure and therapeutic use of these drugs.

**TABLE 3:** Physicochemical properties of the breast cancer drugs.

Drugs	Molar volume (MV) $\left(cm^{3}\right)$	Polarizability (P) $\left(cm^{3}\right)$	Molar refractivity (MR) $\left(cm^{3}\right)$	Polar surface area (PSA) (A°^2^)	Surface tension (ST) (dyne/cm)
Toremifene	367.6	49.1	123.7	12	42.1
Tucatinib	339	53.6	135.2	111	57.3
Ribociclib	311.4	48.9	123.4	91	58.2
Olaparib	301.8	46.3	116.9	82	57.8
Abemaciclib	382.3	55.7	140.4	75	45.8
Anastrozole	270.3	35.7	90	78	42.2
Letrozole	234.5	34.5	87.1	78	53.5
Thiotepa	125.8	19.5	49.1	51	77.8
Tamoxifen	356.2	47.1	118.9	12	40.4
Megestrol Acetate	317.4	40.4	102	60	45.3

### 3.1 Curvilinear regression analysis of drugs for breast cancer

The relationship between a dependent variable (represented by P) and one or more independent variables (represented by RTI) is described by a linear regression model. The independent variables are also called explanatory or predictive variables, and the dependent variable is also called the response variable. In statistical analysis, this model is frequently used to comprehend how one or more independent variables affect the dependent variable. Although this manuscript focuses on linear, quadratic, and cubic regression analysis and its associated parameters, there are other kinds of regression models as well. A variation of linear regression is the quadratic regression and cubic regression model. These model equations are described as follows:

$$P=a_{1}\left(RTI\right)+b$$

$$P=a_{2}{\left(RTI\right)}^{2}+a_{1}\left(RTI\right)+b$$

$$P=a_{3}{\left(RTI\right)}^{3}+a_{2}{\left(RTI\right)}^{2}+a_{1}\left(RTI\right)+b$$

Where RTI is the topological index, b is a constant, $a_{1}$ , $a_{2}$ , $a_{3}$ is the regression coefficient, and P is any of the drugs physicochemical properties. JMP software is used to calculate the constants and coefficients for the molecular structure of drugs, the five physical characteristics of the 10 drugs used to treat breast cancer—molar volume (MV), polarizability (P), molar refractivity (MR), polar surface area (PSA), and surface tension (ST) - are modeled using the RTI mentioned above.

Tables 4-6 present the correlation coefficients (R) obtained from linear, quadratic, and cubic regression models, respectively, highlighting the relationship between resolving topological indices and the physicochemical properties of breast cancer drugs. There are several parameters utilized to retrieve the findings. Tables 7–9 show the linear, quadratic, and cubic regression equations for the greatest fitting and predictability of resolving topological indices, including correlation coefficient value (R), F-statistics, and SE. The correlation coefficient (R) is a statistical metric that describes the strength and direction of a relationship between resolving topological indices and the physicochemical properties. It is expressed as a positive or negative integer between −1 and 1. The number’s value denotes the strength of the association; r = 0 means there is no relationship. All correlation coefficients are more than .7, indicating a significant positive association between the two quantities. The correlation values are negative, indicating an inverse relationship. The p-values measure the strength of the correlation. If the values of p are less than 0.05, the findings of the experiments are significant. Tables show that all resolving topological indices and breast cancer drug features have p-values < 0.001. The p-values indicate the importance of an experiment. The smaller the value of p, the more important the calculations. All computations are significant. The F-value is the ratio of two variances, or mean squares. Regression analysis tests the null hypothesis, which states that all regression coefficients are equal to zero, to establish model significance. The F-value measures the model’s fit and establishes its statistical significance.

**TABLE 4:** The correlation coefficient (R) was obtained utilizing linear regression models.

Resolving topological indices	MV	P	MR	PSA	ST
$FRZI_{1}\left(G\right)$	0.763	0.664	0.665	0.030	**0.736**
$FRZI_{2}\left(G\right)$	**0.896**	**0.903**	**0.903**	**0.178**	0.604
SRZI(G)	0.753	0.670	0.670	0.026	0.692
RHM(G)	0.754	0.671	0.671	0.024	0.693
RF(G)	0.755	0.672	0.672	0.023	0.694

Values highlighted in bold represent the highest correlation for each physicochemical properties and the corresponding topological indices.

**TABLE 5:** The correlation coefficient (R) was obtained utilizing quadratic regression models.

Resolving topological indices	MV	P	MR	PSA	ST
$FRZI_{1}\left(G\right)$	**0.934**	0.885	0.886	0.152	0.799
$FRZI_{2}\left(G\right)$	0.918	**0.903**	**0.903**	**0.291**	**0.870**
SRZI(G)	**0.934**	0.872	0.873	0.029	0.796
RHM(G)	0.933	0.872	0.872	0.030	0.796
RF(G)	0.933	0.871	0.871	0.031	0.797

Values highlighted in bold represent the highest correlation for each physicochemical properties and the corresponding topological indices.

**TABLE 6:** The correlation coefficient (R) was obtained utilizing cubic regression models.

Resolving topological indices	MV	P	MR	PSA	ST
$FRZI_{1}\left(G\right)$	**0.947**	0.887	0.888	0.472	0.80
$FRZI_{2}\left(G\right)$	0.922	**0.906**	**0.905**	**0.511**	**0.871**
SRZI(G)	0.936	0.874	0.875	0.467	0.796
RHM(G)	0.936	0.873	0.874	0.466	0.796
RF(G)	0.935	0.873	0.873	0.465	0.797

Values highlighted in bold represent the highest correlation for each physicochemical properties and the corresponding topological indices.

**TABLE 7:** Linear regression equations offer the most precise estimates of physicochemical properties.

Linear regression equation	R	F	SE	P
$MV=61.071+0.925\left[FRZI_{2}\left(G\right)\right]$	0.896	32.680	35.743	0.0004
$P=8.659+0.1329\left[FRZI_{2}\left(G\right)\right.$	0.903	35.2097	4.948	0.0003
$MR=21.799+0.335\left[FRZI_{2}\left(G\right)\right]$	0.903	35.353	12.462	0.0003
$PSA=44.857+0.078\left[FRZI_{2}\left(G\right)\right]$	0.178	0.261	33.658	0.078
$ST=76.27-0.049\left[FRZI_{1}\left(G\right)\right]$	0.736	9.454	8.222	0.015

**TABLE 8:** Quadratic regression equations offer the most precise estimates of physicochemical properties.

Quadratic regression equation	R	F	SE	P
$MV=-70.494+1.602\left[FRZI_{1}\left(G\right)\right]-0.002{\left[FRZI_{1}\left(G\right)\right]}^{2}$	0.934	23.894	30.799	0.0007
$P=6.389+0.1585\left[FRZI_{2}\left(G\right)\right]-6.1E-05{\left[FRZI_{2}\left(G\right)\right]}^{2}$	0.903	15.477	5.2794	0.0027
$MR=15.875+0.402\left[FRZI_{2}\left(G\right)\right]-0.0002{\left[FRZI_{2}\left(G\right)\right]}^{2}$	0.903	15.545	13.295	0.0027
$PSA=103.12-0.58\left[FRZI_{2}\left(G\right)\right]+0.0016\left[FRZI_{2}\left(G\right)\right]$	0.291	0.323	34.985	0.734
$ST=132.63-0.73\left[FRZI_{2}\left(G\right)\right]+0.002{\left[FRZI_{2}\left(G\right)\right]}^{2}$	0.870	10.871	6.407	0.007

**TABLE 9:** Cubic regression equations offer the most precise estimates of physicochemical properties.

Cubic regression equation	R	F	SE	P
$\begin{array}{cc} MV= & 76.902+0.128\left[FRZI_{1}\left(G\right)\right]+0.002{\left[FRZI_{1}\left(G\right)\right]}^{2}\\ & -3.1E-06{\left[FRZI_{1}\left(G\right)\right]}^{3} \end{array}$	0.947	17.268	29.985	0.002
$\begin{array}{cc} P= & -9.807+0.445\left[FRZI_{2}\left(G\right)\right]-0.002{\left[FRZI_{2}\left(G\right)\right]}^{2}\\ & +2.28E-06{\left[FRZI_{2}\left(G\right)\right]}^{3} \end{array}$	0.906	7.636	6.038	0.026
$\begin{array}{cc} MR= & -15.819+0.948\left[FRZI_{2}\left(G\right)\right]-0.003{\left[FRZI_{2}\left(G\right)\right]}^{2}\\ & +4.13E-06{\left[FRZI_{2}\left(G\right)\right]}^{3} \end{array}$	0.905	9.037	14.260	0.012
$\begin{array}{cc} PSA= & -206.95+4.758\left[FRZI_{2}\left(G\right)\right]-0.025{\left[FRZI_{2}\left(G\right)\right]}^{2}\\ & +4.04E-05{\left[FRZI_{2}\left(G\right)\right]}^{3} \end{array}$	0.511	0.706	33.952	0.582
$\begin{array}{cc} ST= & 119.89-0.511\left[FRZI_{2}\left(G\right)\right]+0.0004{\left[FRZI_{2}\left(G\right)\right]}^{2}\\ & +1.66E-06{\left[FRZI_{2}\left(G\right)\right]}^{3} \end{array}$	0.871	6.292	6.887	0.028

#### 3.1.1 Results

In the linear regression model, $FRZI_{2}\left(G\right)$ has the strongest correlations with MV (R = 0.896), P (R = 0.903), MR (R = 0.903), and PSA (R = 0.178), indicating greater predictive potential among the indices. $FRZI_{1}\left(G\right)$ had the strongest connection with ST (R = 0.736). Indices for SRZI(G) and RHM(G) have slight to almost equal correlations across most properties. These results support the linear model ability to capture linear correlations between resolving topological indices and diverse physicochemical parameters.

In the quadratic regression model, $FRZI_{1}$ (G) and SRZI(G) had the strongest correlation with MV (R = 0.934), showing a robust quadratic association. $FRZI_{2}\left(G\right)$ predicts P (R = 0.903), MR (R = 0.903), PSA (R = 0.291), and ST (R = 0.870), demonstrating its persistent dominance in this model. Meanwhile, RHM(G) and RF(G) produce similar results for all attributes, with only minor differences. The study found that quadratic models outperformed linear models, particularly for indices like $FRZI_{2}\left(G\right)$ .

For the cubic regression model, $FRZI_{1}\left(G\right)$ has the highest correlation with MV (R = 0.947), indicating exceptional predictive strength. It also performs at predicting ST (R = 0.80). $FRZI_{2}\left(G\right)$ has the strongest associations with P (R = 0.906), MR (R = 0.905), and PSA (R = 0.511), indicating its stability across several regression techniques. The indices SRZI(G) and RHM(G) correlate closely, especially in MV, P, and MR. The higher correlation values across all indices indicate that cubic regression models perform better in simulating the link between resolving topological indices and physicochemical properties.

**FIGURE 2:**
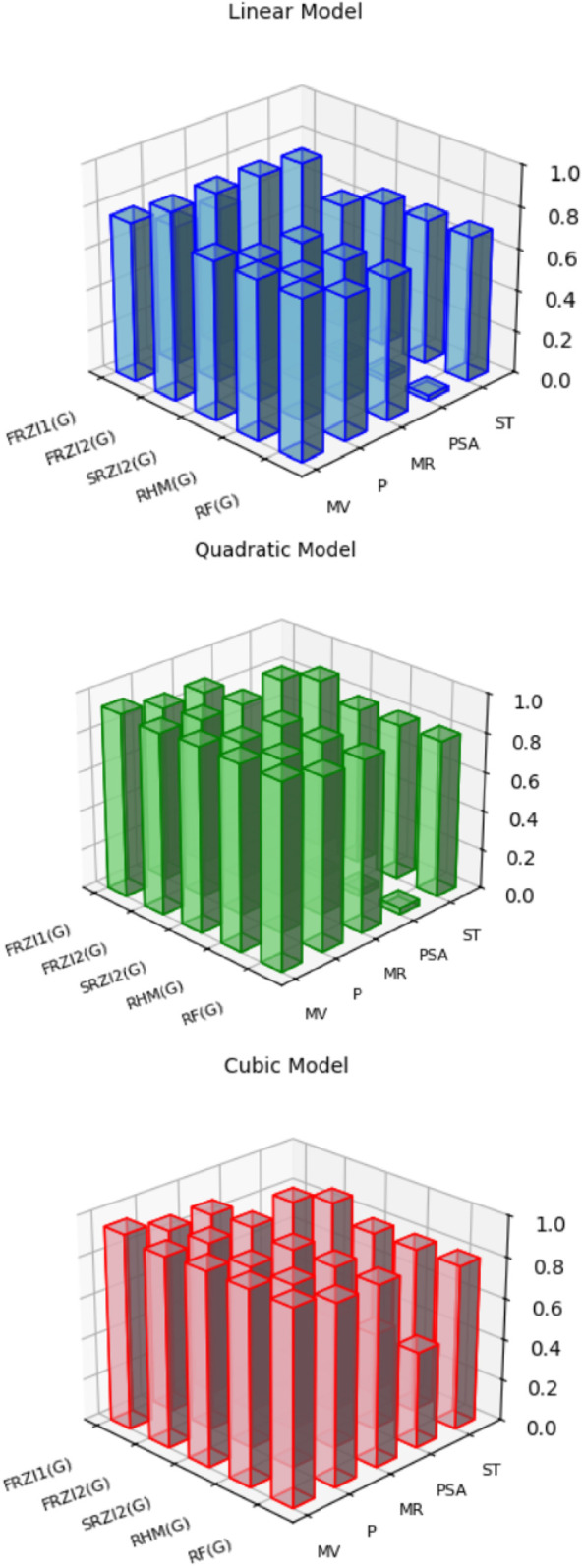
Graphical illustration of the correlation strength between resolving topological indices and physicochemical properties using linear, quadratic, cubic regression analysis.

When linear, quadratic, and cubic regression models are compared, the cubic regression model outperforms them all in terms of predicting physicochemical qualities based on resolving topological indices. As shown in Figure 2, the cubic model regularly produces the greatest correlation coefficients (R), especially for indices such as $FRZI_{1}\left(G\right)$ and $FRZI_{2}\left(G\right)$ . $FRZI_{1}\left(G\right)$ has a high association with MV (R = 0.947), whereas $FRZI_{2}\left(G\right)$ has significant predictive capacity for P, MR, and PSA (R values up to 0.907 and 0.511, respectively). Although the quadratic model outperforms the linear model by capturing certain nonlinear interactions, it is still significantly less accurate than the cubic model. These results show that adding higher-order terms greatly improves the effectiveness of the model. This makes the cubic regression model the best choice for QSPR analysis of breast cancer drugs.

### 3.2 Multiple linear regression model

Multiple linear regression is a statistical approach for examining the connection between a dependent variable and several independent variables, modeling how predictors impact the outcome, and quantifying their effects.

Using the Variance Inflation Factor (VIF), multicollinearity among the chosen topological descriptors was assessed in all MLR models. Multicollinearity occurs when two or more independent variables in a regression model are strongly correlated, affecting the predicted coefficients. The Variance Inflation Factor (VIF) detects multicollinearity and is computed as:

$${\text{VIF}}_{i}=\frac{1}{1-R_{i}^{2}}$$

where ${\text{VIF}}_{i}$ is the VIF for the i -th independent variable $X_{i}$ , and $R_{i}^{2}$ is the coefficient of determination obtained when $X_{i}$ is regressed against all other independent variable. Multicollinearity values ( < 10) are considered acceptable. In the present study, all descriptors obtained VIF values ranging from 4.8 to 5.6, suggesting no significant multicollinearity. The main MLR equation is

$$Y=\alpha_{0}+\alpha_{1}X_{1}+\alpha_{2}X_{2}+\cdots +\alpha_{p}X_{p}$$ (11)

where Y is the dependent variable, $X_{1},X_{2},\ldots ,X_{p}$ are the independent variables, and $\alpha_{1},\alpha_{2},\ldots ,\alpha_{p}$ are the regression coefficients. The intercept, or regression constant, is denoted as $\alpha_{0}$ . Each coefficient shows the change in Y for a one-unit increase in the related predictor, while leaving other variables constant. This demonstrates that each topological descriptor makes an independent contribution to the prediction of the observed physicochemical properties. Using Equation 11, the multiple linear regression models corresponding to the resolving topological indices analyzed in this study are derived as follows.

$$MV=57.8378-0.0732\left[FRZI_{1}\left(G\right)\right]+1.0778\left[FRZI_{2}\left(G\right)\right],$$

R = 0.90, $R^{2}$ = 0.81, SE = 37.644, F = 14.838, Significant = 0.003.

$$P=6.2646+0.2428\left[FRZI_{2}\left(G\right)\right]-0.0116\left[RHM\left(G\right)\right],$$

R = 0.968, $R^{2}$ = 0.94, SE = 3.1067, F = 51.3009, Significant = 0.0001.

$$MR=17.135-0.1057\left[FRZI_{1}\left(G\right)\right]+0.556\left[FRZI_{2}\left(G\right)\right]$$

R = 0.953, $R^{2}$ = 0.91, SE = 9.3815, F = 34.7487, Significant = 0.0002.

$$PSA=38.1582+0.3772\left[FRZI_{2}\left(G\right)\right]-0.1264\left[SRZI\left(G\right)\right]$$

R = 0.365, $R^{2}$ = 0.134, SE = 34.032, F = 0.54, Significant = 0.605.

$$ST=73.5937-0.063\left[FRZI_{1}\left(G\right)\right]+0.0379\left[FRZI_{2}\left(G\right)\right],$$

R = 0.744, $R^{2}$ = 0.55, SE = 8.6705, F = 4.3480, Significant = 0.05.

#### 3.2.1 Results of multiple linear regression (MLR) analysis

The multiple linear regression (MLR) model was created to study the connection between the dependent variable and the chosen resolving topological indices.

• The MLR model for molar volume showed a strong fit ( $R^{2}=0.809$ , R=0.90 ), explaining approximately 80% of the variance. The overall model was statistically significant ( F=14.84 , p<0.05 ). The descriptor $FRZI_{2}\left(G\right)$ had a substantial favorable effect ( p=0.0235 ), whereas $FRZI_{1}\left(G\right)$ contributed negatively but insignificantly ( p=0.6589 ). Variance Inflation Factor (VIF) scores ( < 10) indicated the absence of multicollinearity. Thus, $FRZI_{2}\left(G\right)$ is crucial for predicting the molar volume of the molecules under study.

• Polarizability demonstrated a high correlation ( $R^{2}=0.936$ , R=0.968 ) and substantial model significance ( F=51.30 , p<0.05 ). The descriptor $FRZI_{2}\left(G\right)$ had a substantial favorable effect ( p=0.0002 ), whereas RHM(G) had a significant negative impact ( p=0.0082 ). VIF values ( ∼5.6 ) indicated no multicollinearity. The low RMSE (3.10) compared to the mean response (43.08) demonstrated strong predictive accuracy. Thus, $FRZI_{2}\left(G\right)$ and RHM(G) together form an effective model for predicting polarizability.

• The MLR model for molar refractivity (MR) demonstrated a significant correlation ( $R^{2}=0.908$ , R=0.882 ) and high model significance ( F=34.75 , p<0.05 ). The descriptor $FRZI_{2}\left(G\right)$ had a substantial positive effect ( p=0.0006 ), while $FRZI_{1}\left(G\right)$ exhibited a significant negative influence ( p=0.0321 ). VIF values ( ∼4.8 ) indicated no multicollinearity. The RMSE (9.38) relative to the mean response (108.67) demonstrated high predictive ability. Both $FRZI_{1}\left(G\right)$ and $FRZI_{2}\left(G\right)$ explain variations in molar refractivity, although $FRZI_{2}\left(G\right)$ is the primary contributor. Overall, the model shows that molecular connectivity indices are strongly correlated with molar refractivity, highlighting their applicability in QSPR studies.

• In contrast, the polar surface area (PSA) showed a poor correlation ( $R^{2}=0.134$ , R=0.365 ) and was not statistically significant ( F=0.54 , p>0.05 ). Both $FRZI_{2}\left(G\right)$ ( p=0.3344 ) and SRZI(G) ( p=0.3939 ) did not significantly contribute to PSA prediction, indicating weak descriptor relevance. The comparatively large RMSE (34.03) relative to the mean response (65) indicates low predictive accuracy. Although VIF values ( ∼5.6 ) suggested negligible multicollinearity, the model’s overall performance was unsatisfactory. This suggests that PSA may be influenced by other molecular characteristics, such as hydrogen bonding capacity, polar functional groups, or surface topology, which are not fully captured by the descriptors used.

• The surface tension (ST) demonstrated a moderate correlation ( $R^{2}=0.554$ , R=0.744 ) with limited overall significance ( F=4.35 , p>0.05 ). The descriptors $FRZI_{2}\left(G\right)$ ( p=0.6727 ) and $FRZI_{1}\left(G\right)$ ( p=0.1288 ) had modest effects, indicating limited predictive influence on surface tension. The RMSE (8.67) relative to the mean response (52.04) suggests reasonable prediction accuracy, while VIF values ( ∼4.8 ) reveal no multicollinearity. Overall, the model explains only a portion of the variance in surface tension, suggesting that additional structural or intermolecular descriptors may be needed for improved prediction.

**FIGURE 3:**
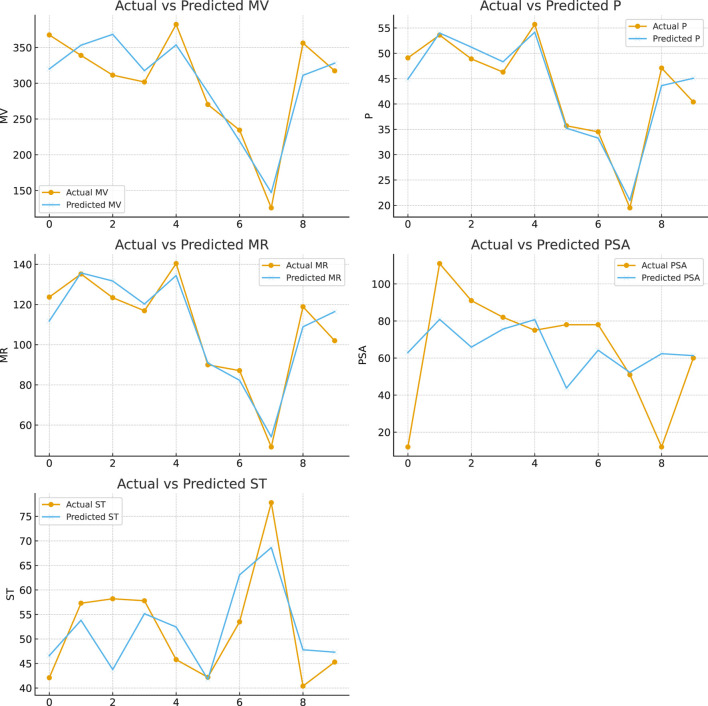
Plots of predicted values of resolving topological indices and MV, P, MR, PSA and ST from MLR analysis.

These findings emphasize the distinct contributions of each descriptor to the model. Figure 3 shows predicted values for resolving topological indices and molecular descriptors such as MV, P, MR, PSA, and ST obtained from the MLR study, demonstrating the relationship between these variables and the model’s prediction accuracy.

## 4 Discussion

We used linear, quadratic, and cubic regression models to see how well resolving topological indices could predict future events in this study. The results showed that $FRZI_{2}\left(G\right)$ had the highest correlation coefficients for a number of physicochemical properties, especially molar refractivity (MR) and polarizability (P). The fact that these results are consistent across model types suggests that $FRZI_{2}\left(G\right)$ does a good job of demonstrating significant molecular features. Other indices, such as SRZI(G) , RHM(G) , and RF(G) , were able to make predictions with moderate accuracy. The results support the use of resolving indices in QSPR modeling for drugs that treat breast cancer. However, the study has some problems because it only used a small dataset and regression analysis. Future research could look into machine learning models and try these descriptors on bigger drug databases that also include biological endpoints. The MLR analysis indicated that the selected topological descriptors had various resolving degrees of effect on the physicochemical properties under study. Descriptors like $FRZI_{2}$ were highly predictive for qualities including molar volume, polarizability, and molar refractivity, whereas $FRZI_{1}$ and SRZI exhibited minimal influence. Multicollinearity was minimal, showing that each descriptor makes an independent contribution to the models. The present descriptors offered limited prediction accuracy for features such as polar surface area and surface tension, indicating the need for new molecular parameters. Overall, our findings demonstrate the utility of resolving topological indices for representing some physicochemical properties while underlining the necessity for more extensive descriptors for others. The strong correlation of $FRZI_{2}$ with P and MR can be explained by the index’s sensitivity to differences in molecular connectivity and bond distribution, which affect electron delocalization and, as a result, molecule polarizability and refractivity. In contrast, PSA poor predictive ability might be attributed to the fact that PSA’s predominantly determined by the quantity and orientation of polar functional groups, whereas the topological indices utilized in this work represent global structural aspects rather than local polarity effects.

To evaluate the predictive power of each model in simulating the link between resolving topological indices and physicochemical properties, the performance of linear, quadratic, cubic, and multiple linear regression (MLR) models was examined (Table 10). All things considered, the comparison demonstrates that MLR is the best modeling strategy for this dataset, whereas PSA plays an insignificant role in drug activity estimates. In the case of PSA, the regression model demonstrated less statistical significance (p > 0.05), indicating a limitation of the current study. This might be attributable to the small dataset size, which limits the statistical power of the analysis. However, other models, such as MV and MR, demonstrated substantial significance and predictive ability, indicating that resolving indices are generally good descriptors. This conclusion shows that, while the technique is promising, more validation with bigger and more varied datasets will be necessary to strengthen weaker models and improve generalizability.

**TABLE 10:** Comparative results of linear, quadratic, cubic, and MLR models for molecular properties.

Properties	LR (R, p < 0.05)	QR (R, p < 0.05)	CR (R, p < 0.05)	MLR (R, p < 0.05)
MV	0.896, < 0.0004	0.934, < 0.0007	0.947, < 0.002	0.900, < 0.05
P	0.903, < 0.0003	0.903, < 0.0027	0.906, < 0.026	0.968, < 0.05
MR	0.903, < 0.0003	0.903, < 0.0027	0.905, < 0.012	0.953, < 0.05
PSA	0.178, > 0.078	0.291, > 0.734	0.511, > 0.582	0.366, > 0.05
ST	0.736, < 0.015	0.870, < 0.007	0.871, < 0.028	0.744, > 0.05

Several research studies have investigated the use of topological indices to predict the physicochemical features of breast cancer drugs. For example, standard degree-based topological indices were used for breast cancer drugs and found strong correlations with certain physicochemical properties [Bokhary et al., 2022; Shanmukha et al., 2022; Meharban et al., 2024). Entire neighborhood topological indices were then developed, using cubic and multiple regression approaches, and these exhibited better relationships with drug attributes (Altassan et al., 2025). CoM-polynomial-based indices were also examined, computing variable topological coindices, and several indices showed significant predictive capacity using curvilinear regression analysis (Öztürk Sözen and Eryaşar, 2024). More recently, entropy-based indices were developed using both linear and cubic regression techniques, and specific entropy indices were found to substantially predict attributes such as boiling point, molar volume, and melting point (Rauf et al., 2023b). While these studies demonstrate the adaptability of topological indices in QSPR modeling of breast cancer drugs, the majority use standard degree-based indices, neighborhood indices, or entropy-based indices. In contrast, the current study highlights the use of resolving topological indices, which offer a new perspective by merging structural uniqueness and molecular symmetry into the characterization of chemical graphs. This technique adds a new dimension to QSPR research, potentially improving predicted accuracy and offering more insight into drug features. Compared to previous research, our work broadens the field of topological index applications by looking at the efficacy of resolving indices for breast cancer drugs. The findings suggest that resolving indices may be useful descriptors in chemical graph theory, supplementing and expanding the predictive power of previously examined indices.

## 5 Conclusion

In this study, we used both linear and multiple linear regression (MLR) models to examine the relationship between resolving topological indices and important physicochemical properties of drugs used to treat breast cancer. According to the results of linear regression, indices like $FRZI_{2}\left(G\right)$ consistently generated high correlation coefficients, especially with molar refractivity (MR) and polarizability (P), indicating the predictive power of each one alone. Out of all the indices $FRZI_{2}\left(G\right)$ had the highest correlation of R = 0.906 in the cubic regression model, showing its adaptability to changes in model complexity. By combining several indices as predictors, the multiple linear regression (MLR) model, on the other hand, provided a more thorough evaluation. The models exceptional performance in predicting MV ( R = 0.900), P ( R = 0.968) and MR ( R = 0.953) suggests that the combination of indices greatly improves prediction accuracy. The models performance declined for PSA, though ( R = 0.366), indicating that the chosen indices had little predictive value for this specific properties. For some properties, MLR can capture moderate to strong relationships as evidenced by the comparatively high R for ST (0.744). All things considered, the results show that resolving topological indices have a great deal of promise for simulating the physicochemical properties of breast cancer drugs, particularly when combined via MLR. The models created in this study have the potential to improve the effectiveness of molecular design and drug screening procedures. To improve prediction accuracy, future studies can broaden this methodology to incorporate more molecular descriptors and advanced machine learning algorithms, including Back Propagation Neural Networks (BPNN), GA-BPNN, and Support Vector Regression (SVR), which are adept at modeling difficult and nonlinear relationships between topological indices and physicochemical properties (Zonghuang, 2023; Karampuri and Perugu, 2024; Chang et al., 2013).

### 5.1 Implications

Drug activity prediction for breast cancer may be enhanced by QSPR modeling and resolving topological indices, enabling safer and more efficient treatment approaches. Understanding molecular descriptors can help pharmacists and chemists optimize medication discovery and design, which will ultimately result in more individualized and accurate cancer treatments.

### 5.2 Limitation

The primary limitation of this study is the limited dataset of ten breast cancer drugs, which constrains the generalizability of the regression findings. The limited sample size may not accurately represent the extensive chemical and therapeutic diversity of breast cancer treatments. Nonetheless, the selected drugs were incorporated due to the availability of reliable experimental data and their significance as clinically important therapies. Although the models offer valuable insights into the correlation between resolving topological indices and drug activity, it is crucial to expand the dataset in future studies to enhance robustness, validate findings, and improve predictive accuracy.

## Data Availability

The original contributions presented in the study are included in the article/supplementary material, further inquiries can be directed to the corresponding author.

## References

[B1] Altassan A. Saleh A. Alashwali H. Hamed M. Muthana N. (2025). Exploring qspr in breast cancer drugs *via* entire neighborhood indices and regression models. Sci. Rep. 15, 26683. 10.1038/s41598-025-12179-0 40696006 PMC12284210

[B2] Arockiaraj M. Godlin J. J. Radha S. (2025). Comparative study of degree and neighborhood degree sum-based topological indices for predicting physicochemical properties of skin cancer drug structures. Mod. Phys. Lett. B 39, 2550106. 10.1142/S0217984925501064

[B3] Bokhary S. A. U. H. Siddiqui M. K. Cancan M. (2022). On topological indices and qspr analysis of drugs used for the treatment of breast cancer. Polycycl. Aromat. Compd. 42, 6233–6253. 10.1080/10406638.2021.1977353

[B4] Bommahalli Jayaraman B. Siddiqui M. K. (2024). Exploring the properties of antituberculosis drugs through qspr graph models and domination-based topological descriptors. Sci. Rep. 14, 24387. 10.1038/s41598-024-73918-3 39420019 PMC11487200

[B5] Chang Y.-H. Chen J.-Y. Hor C.-Y. Chuang Y.-C. Yang C.-B. Yang C.-N. (2013). Computational study of estrogen receptor-alpha antagonist with three-dimensional quantitative structure-activity relationship, support vector regression, and linear regression methods. Int. J. Med. Chem. 2013, 1–13. 10.1155/2013/743139 25505989 PMC4245501

[B6] Gutman I. Trinajstić N. (1972). Graph theory and molecular orbitals. Total *φ*-electron energy of alternant hydrocarbons. Chem. Phys. Lett. 17, 535–538. 10.1016/0009-2614(72)85099-1

[B7] Hakeem A. Ullah A. Zaman S. Mahmoud E. E. Ahmad H. Ali P. (2025). Topological modeling and qspr based prediction of physicochemical properties of bioactive polyphenols. Sci. Rep. 15, 27466. 10.1038/.s41598-025-11863-5 40721442 PMC12304092

[B8] Harary F. Melter R. A. (1976). On the metric dimension of a graph. Ars Comb. 2, 1.

[B9] Havare Ö. Ç. (2021). Topological indices and qspr modeling of some novel drugs used in the cancer treatment. Int. J. Quantum Chem. 121, e26813. 10.1002/qua.26813

[B10] Kara Y. Özkan Y. S. Arockiaraj M. (2025a). Computational insights and predictive models for lung cancer molecular structures. Chem. Pap. 79, 1869–1878. 10.1007/s11696-025-03894-z

[B11] Kara Y. Özkan Y. S. Ullah A. Hamed Y. S. Belay M. B. (2025b). Qspr modeling of some covid-19 drugs using neighborhood eccentricity-based topological indices: a comparative analysis. PLoS One 20, e0321359. 10.1371/journal.pone.0321359 40392879 PMC12091765

[B12] Karampuri A. Perugu S. (2024). A breast cancer-specific combinational qsar model development using machine learning and deep learning approaches. Front. Bioinforma. 3, 1328262. 10.3389/fbinf.2023.1328262 38288043 PMC10822965

[B13] Kirmani S. A. K. Ali P. Azam F. (2021). Topological indices and qspr/qsar analysis of some antiviral drugs being investigated for the treatment of covid-19 patients. Int. J. Quantum Chem. 121, e26594. 10.1002/qua.26594 33612855 PMC7883265

[B14] Kour S. J. R. S. (2024). Machine learning regression models for predicting anti-cancer drug properties: insights from topological indices in qspr analysis. Contemp. Math., 6515–6526doi. 10.37256/cm.5420245826

[B15] Kour S. Ravi Sankar J. (2025). Hydrogen-centric machine learning approach for analyzing properties of tricyclic anti-depressant drugs. Front. Chem. 13, 1603948. 10.3389/fchem.2025.1603948 40529530 PMC12170657

[B16] Kour S. Sankar J. R. (2025). Characterization of tricyclic anti-depressant drugs efficacy *via* topological indices. Sci. Rep. 15, 22853. 10.1038/s41598-025-05045-6 40594093 PMC12217923

[B17] Kumar V. Das S. (2024). Comparative study of gq and qg indices as potentially favorable molecular descriptors. Int. J. Quantum Chem. 124, e27334. 10.1002/qua.27334

[B18] Liu J. B. (2022). Novel applications of graph theory in chemistry and drug designing. Comb. Chem. and High Throughput Screen. 25, 439–440. 10.2174/1386207325666220104223136 35038980

[B19] Liu J.-B. Singaraj R. M. (2021). Topological analysis of para-line graph of remdesivir used in the prevention of corona virus. Int. J. Quantum Chem. 121, e26778. 10.1002/qua.26778

[B20] Mahboob A. Rasheed M. W. Hanif I. Amin L. Alameri A. (2024). Role of molecular descriptors in quantitative structure-property relationship analysis of kidney cancer therapeutics. Int. J. Quantum Chem. 124, e27241. 10.1002/qua.27241

[B21] Meharban S. Ullah A. Zaman S. Hamraz A. Razaq A. (2024). Molecular structural modeling and physical characteristics of anti-breast cancer drugs *via* some novel topological descriptors and regression models. Curr. Res. Struct. Biol. 7, 100134. 10.1016/j.crstbi.2024.100134 38516623 PMC10955308

[B22] Öztürk Sözen E. Eryaşar E. (2024). An algebraic approach to calculate some topological coindices and qspr analysis of some novel drugs used in the treatment of breast cancer. Polycycl. Aromat. Compd. 44, 2226–2243. 10.1080/10406638.2023.2214286

[B23] Pandeeswari E. Ravi Sankar J. (2025). Investigating metric and edge metric resolvability in molecular structures of breast cancer therapeutics. Malays. J. Math. Sci. 19, 1079–1110. 10.47836/mjms.19.3.16

[B24] Pandeeswari E. Sankar J R. (2025). Computational approaches to predict nsaid characteristics using degcity indices and qspr analysis. Contemp. Math., 1331–1346doi. 10.37256/cm.6120255205

[B25] Randic M. (1975). Characterization of molecular branching. J. Am. Chem. Soc. 97, 6609–6615. 10.1021/ja00856a001

[B26] Rao Y. Chen R. Ahmad H. Ahmad U. (2024). Reverse zagreb indices and their application in the evaluation of physiochemical properties of anticancer/antibacterial drugs. ACS omega 9, 31056–31080. 10.1021/acsomega.4c04409 39035877 PMC11256078

[B27] Rauf A. Naeem M. Hanif A. (2023a). Quantitative structure–properties relationship analysis of eigen-value-based indices using covid-19 drugs structure. Int. J. Quantum Chem. 123, e27030. 10.1002/qua.27030 36718482 PMC9877715

[B28] Rauf A. Naeem M. Rahman J. Saleem A. V. (2023b). Qspr study of ve-degree based end vertice edge entropy indices with physio-chemical properties of breast cancer drugs. Polycycl. Aromat. Compd. 43, 4170–4183. 10.1080/10406638.2022.2086272

[B29] Sardar M. S. Hakami K. H. (2024). Qspr analysis of some alzheimer’s compounds *via* topological indices and regression models. J. Chem. 2024, 5520607. 10.1155/2024/5520607

[B30] Shanmukha M. Usha A. Praveen B. Douhadji A. (2022). Degree-Based molecular descriptors and QSPR analysis of breast cancer drugs. J. Math. 2022, 5880011. 10.1155/2022/5880011

[B31] Slater P. (1975). Leaves of trees. Congr. Numerantium.

[B32] Sooryanarayana B. Chandrakala S. B. Roshini G. R. Kumar M. V. (2022). Resolving topological indices of graphs. Iran. J. Math. Chem. 13, 201–226. 10.22052/ijmc.2022.242888.1567

[B33] P U. P. Suresh M. Tolasa F. T. Bonyah E. (2024). Qspr/Qsar study of antiviral drugs modeled as multigraphs by using ti’s and mlr method to treat covid-19 disease. Sci. Rep. 14, 1–14. 10.1038/s41598-024-63007-w 38849399 PMC11711684

[B34] Zonghuang X. (2023). Machine learning-based quantitative structure-activity relationship and admet prediction models for er*α* activity of anti-breast cancer drug candidates. Wuhan Univ. J. Nat. Sci. 28, 257–270. 10.1051/wujns/2023283257

